# Supplementary material for: Decoupling tRNA promoter and processing activities enables specific Pol-II Cas9 guide RNA expression
Source: Nat Commun. 2019 Apr 2;10:1490. doi: 10.1038/s41467-019-09148-3 (PMC6445147; doi:10.1038/s41467-019-09148-3)
Supplement: Supplementary file 1 — Supplementary Information [file 41467_2019_9148_MOESM1_ESM.pdf]

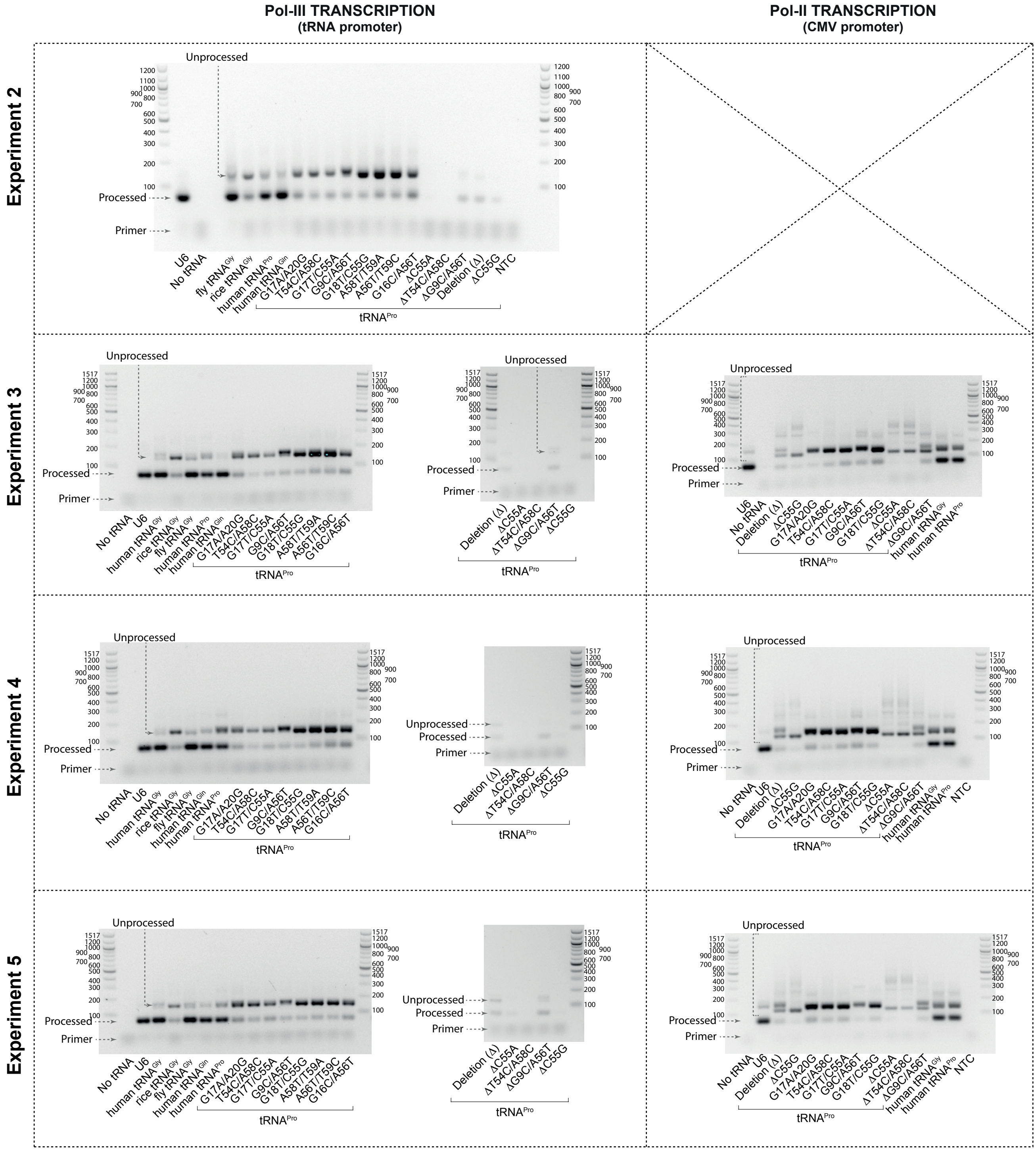

Supplementary Figure 1. Agarose gel images used to determine processing efficiency. Gel images are shown for each experiment in which the RNA circularization assay was performed. Images from constructs with no external promoter are shown in the left column and those which had a Pol-II (CMV) promoter on the right. For Pol-II promoter containing assays RNA was first dephosphorylated prior to circularization. Size in base pairs (bp) are shown for all ladders. The approximate location of successfully processed bands, unprocessed bands, and residual primer are noted on each gel. Saturated pixels (where present) are shown in green.

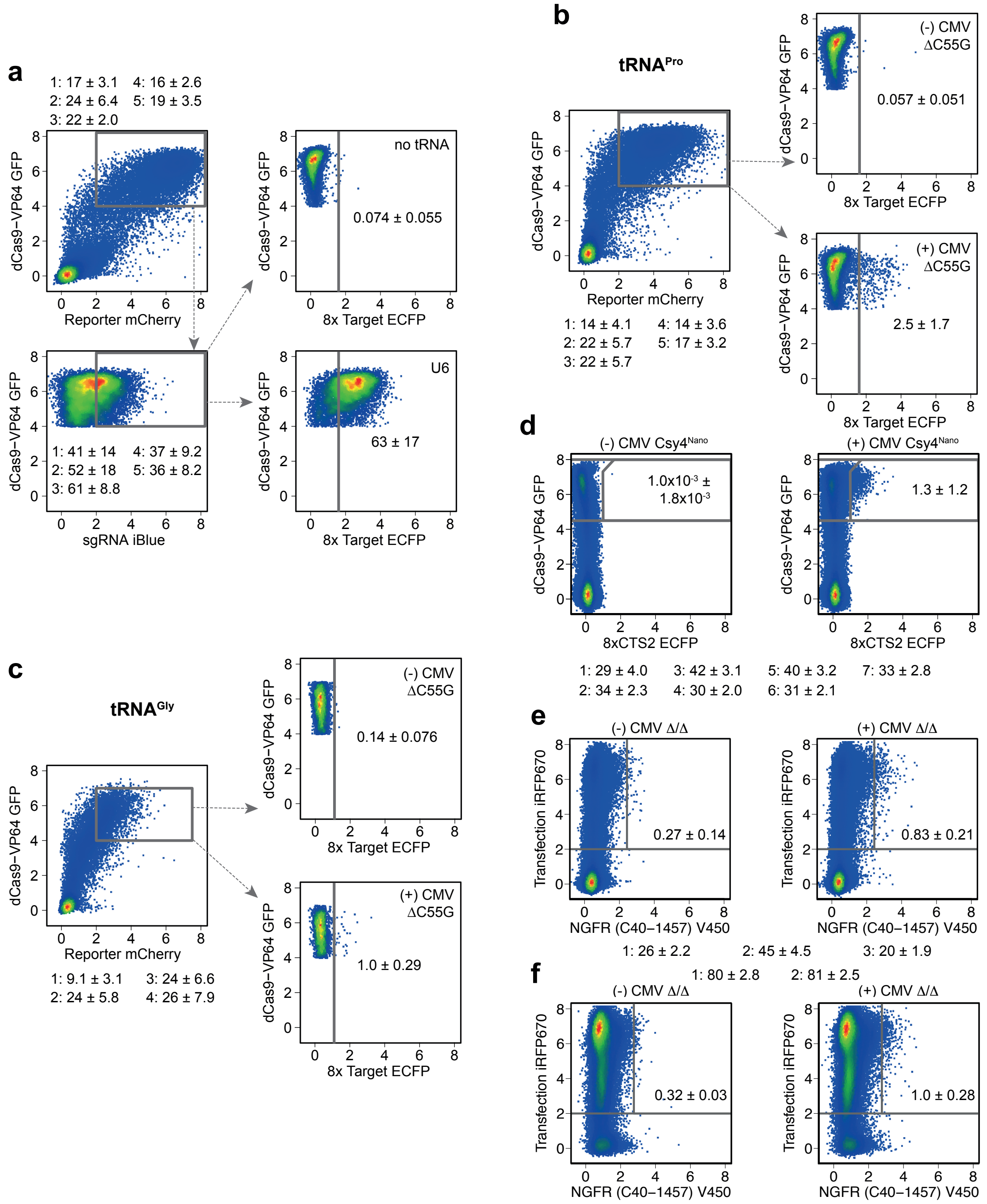

Supplementary Figure 2. Representative gating strategies used in the manuscript. (a) Gating hierarchy used in all $tRNA^{Pro}$ promoter testing experiments. Axes represent the asinh($ECFP_{fluoresence}$/150) of the fluorescence measurement (similar to a biexponential transformation). Values shown are mean ± s.d. for each experiment for the first two gates, and the mean ± s.d. across experiments for the final gate for the two examples shown. Plasmids expressed EGFP (dCas9-VP64), mCherry (in the reporter backbone), or iBlue (in the tRNA-sgRNA plasmids). Final ECFP gating was performed on cells positive for all requisite plasmids. In some cases, a maximum threshold was set on EGFP fluorescence where spillover would have been an issue. **(b, c)** Example of gating strategy for ON-OFF ratio used for $tRNA^{Pro}$ **(b)** and for $tRNA^{Gly}$ **(c)**. Mean ± s.d is shown per experiment for the first gate, and for the subsequent example final gates the mean ± s.d. across replicates is shown. In these instances, iBlue gating was not used in the gRNA plasmids as preliminary experiments suggested a crosstalk with the CMV promoter. **(d)** Gating strategy for alternative methods of gRNA processing. In this case only the EGFP on dCas9-VP64 was retained as other colors conflicted with some used in the alternative strategies. Mean ± s.d. % $EGFP^{+}$ are shown for each experiment. Mean ± s.d. % $ECFP^{+}$ (of $EGFP^{+}$) are shown across replicates for the selected examples. **(e, f)** Example gating for NGFR activation experiments without antibiotic selection 3 days after transfection **(e)** and with selection 4 days after transfection **(f)**. Mean ± s.d. % $iRFP670^{+}$ (transfection control positive) are shown for each experiment. Mean ± s.d. % $NGFR^{+}$ (of $iRFP670^{+}$) are shown across replicates for the selected examples.

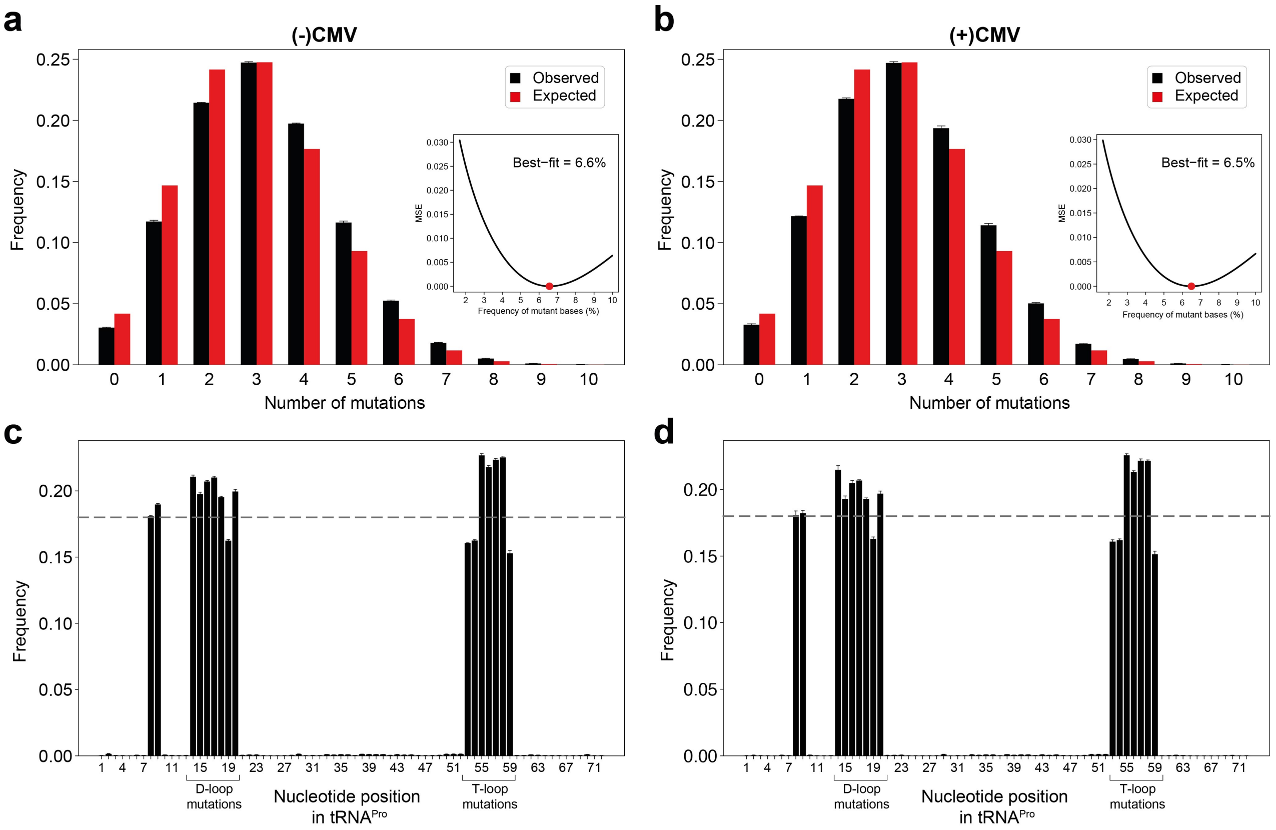

Supplementary Figure 3. Mutation frequency and location in the tRNA screening libraries. (a, b) Frequencies for each number of mutations observed in the plasmid library without **(a)** and with **(b)** CMV promoters. The expected frequency based on the 6% of each non-WT base in the ordered oligo pool is shown in red. Black bars represent the frequency across three biological replicates for each library (mean ± s.d.). Insets show the mean-squared error (m.s.e.) of the measured mutational frequency compared to expected with differing frequencies of non-WT bases. Optimal fits are shown by red points and indicated in text. Best fits (minimum m.s.e.) are estimated at 6.6% and 6.5% without and with CMV promoters respectively, which closely match the 6% in the ordered pool. These results suggest that the diversity was not bottlenecked at any point in the protocol. **(c, d)** The frequency of mutations by location in the tRNA is shown for plasmid libraries without **(c)** or with **(d)** CMV promoters (n = 3 biological replicates, mean ± s.d.). These results confirm that mutant bases are specific for the targeted nucleotides. The expected overall mutation rate of 18% (given 6% of each non-WT base) is shown as a dotted line. Source data are provided as a Source Data file.

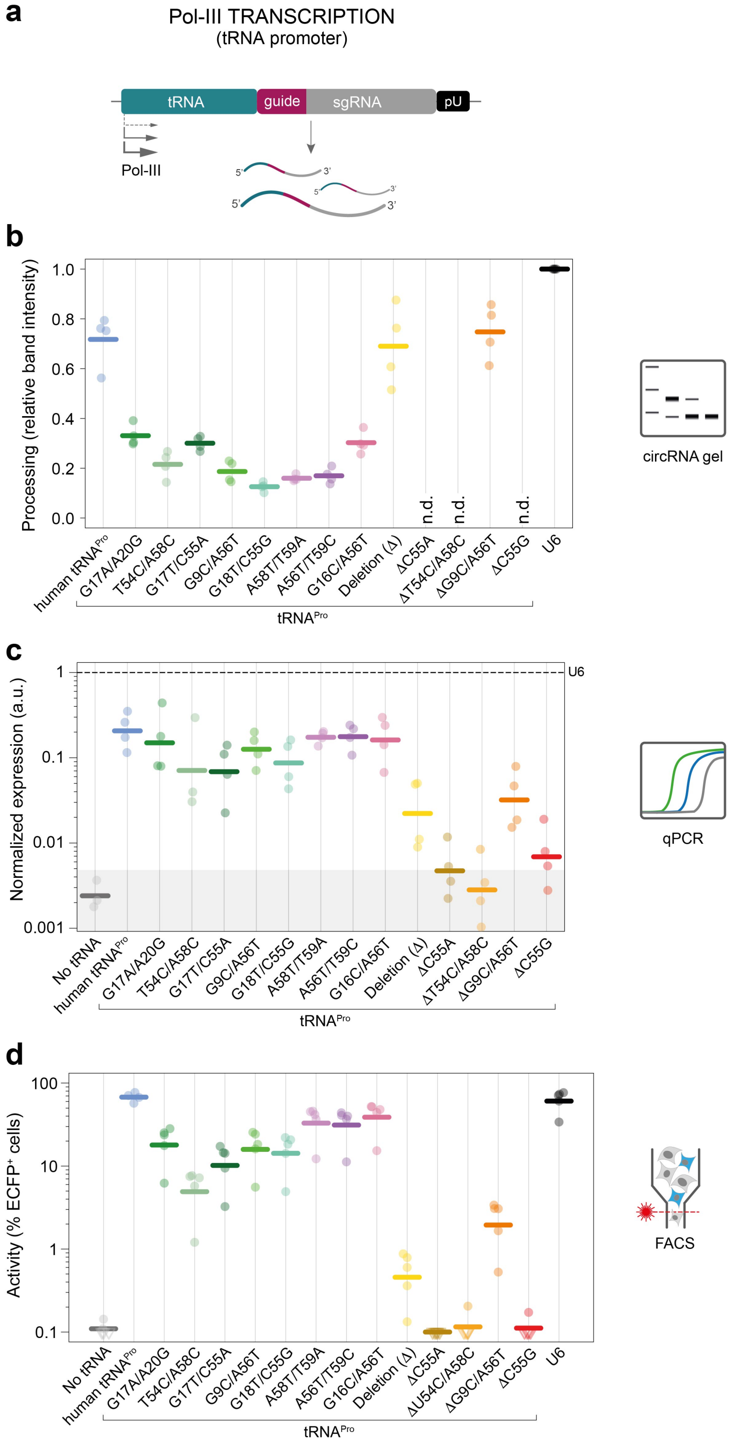

Supplementary Figure 4. Characterization of promoter, 3' processing and background gRNA production of engineered tRNA scaffolds. (a) Constructs and resulting RNA used for testing Pol-III promoter activity. **(b)** 3' processing ability of each mutant tRNA tested (n = 4 independent experiments). Wild-type tRNA^{Pro} and U6 controls are shown for reference from Fig. 1d. Efficiency represents the ratio of band intensity between the unprocessed and processed bands on a 2% agarose gel following RNA circularization and nested RT-PCR (thick lines = mean values; ND = variants with no detectable bands). All double-mutants showed some loss of 3' processing compared to wild-type > 95% probability (paired BEST test). **(c)** gRNA expression as measured by qPCR relative to Cas9 and U6 (ΔΔCt) (n = 4 independent experiments, n = 3 for no promoter control; dashed line = gRNA levels for U6) (U6, 'no tRNA', human tRNA^{Pro} and tRNA^{Gly} are shared with Fig. 1c). Shaded area represents the 75% credible mass (BEST test) for the no tRNA control. The T54C/A58C, G17T/C55A and G18T/C55G double-mutants predicted to decrease promoter activity (green tones) had a >80% probability, and all ΔtRNA scaffolds showed 95% probability of decreased promoter activity compared to wild-type (paired BEST tests). **(d)** Percent reporter ECFP^{+} cells within all transfected cells (thick lines = geometric mean values; each point = independent experiment; n = 4-5). U6, wild-type tRNA^{Pro}, and no promoter controls are shared with Fig. 1e, f, and 3d, e. (hollow downward triangles = points at or below the limit of detection). Double-mutants affecting only processing (pink tones) had somewhat decreased functional activity compared to wild-type 99% probability, while those affecting promoter activity were even lower with >99% probability of decrease compared to all processing double-mutants (paired BEST tests). Source data are provided as a Source Data file.

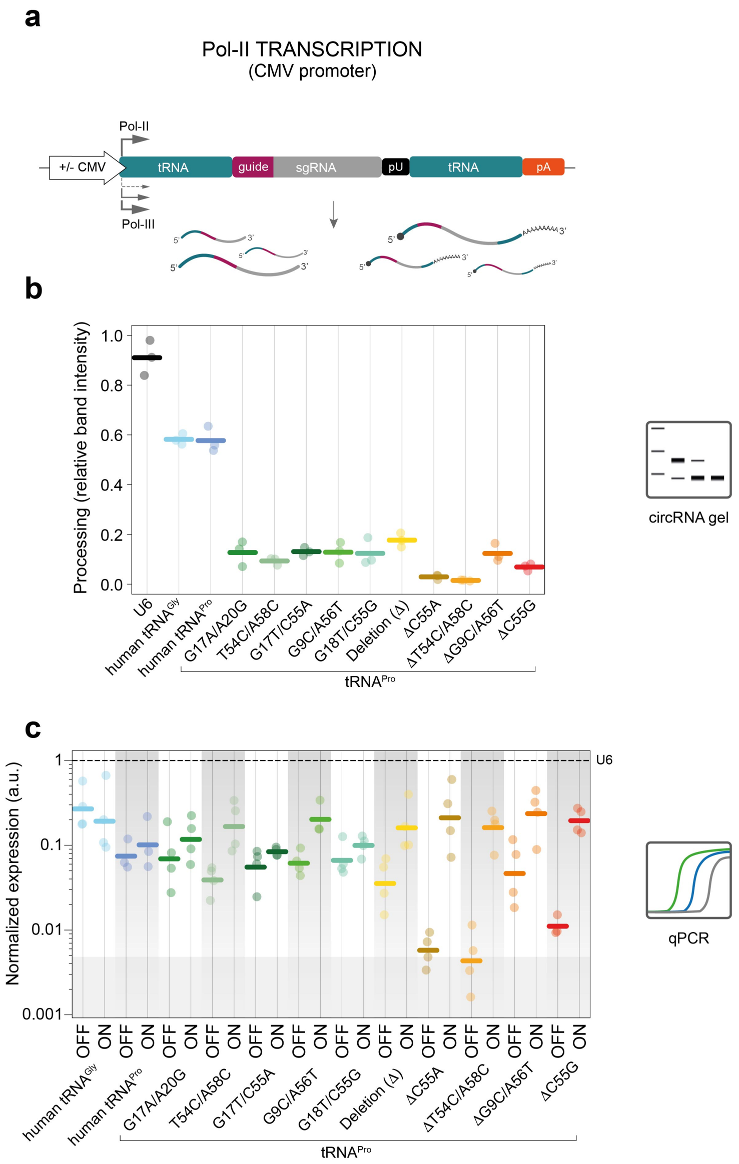

Supplementary Figure 5. Characterization of engineered tRNA scaffolds overall processing and ON/OFF ratios in the presence and absence of Pol-II promoter. (a) Diagram of constructs used to test ON/OFF ratios in presence and absence of a Pol-II promoter (shared with Fig. 3c). **(b)** Overall processing ability of each mutant tRNA tested (n = 4 independent experiments). Efficiency represents the ratio of band intensity between the processed band and all other bands in the lane on a 2% agarose gel following decapping, RNA circularization and nested RT-PCR (thick lines = mean values). **(c)** gRNA expression for each mutation in the presence (ON) and absence (OFF) of a Pol-II promoter (CMV) as measured by qPCR relative to Cas9 and U6 (ΔΔCt) (n = 4 independent experiments, n = 3 for no promoter control; dashed line = gRNA levels for U6) (U6 and 'no tRNA' are shared with Fig. 1c and Supplementary Fig. 4c). Shaded area represents the 75% credible mass (BEST test) for the no tRNA control. Source data are provided as a Source Data file.

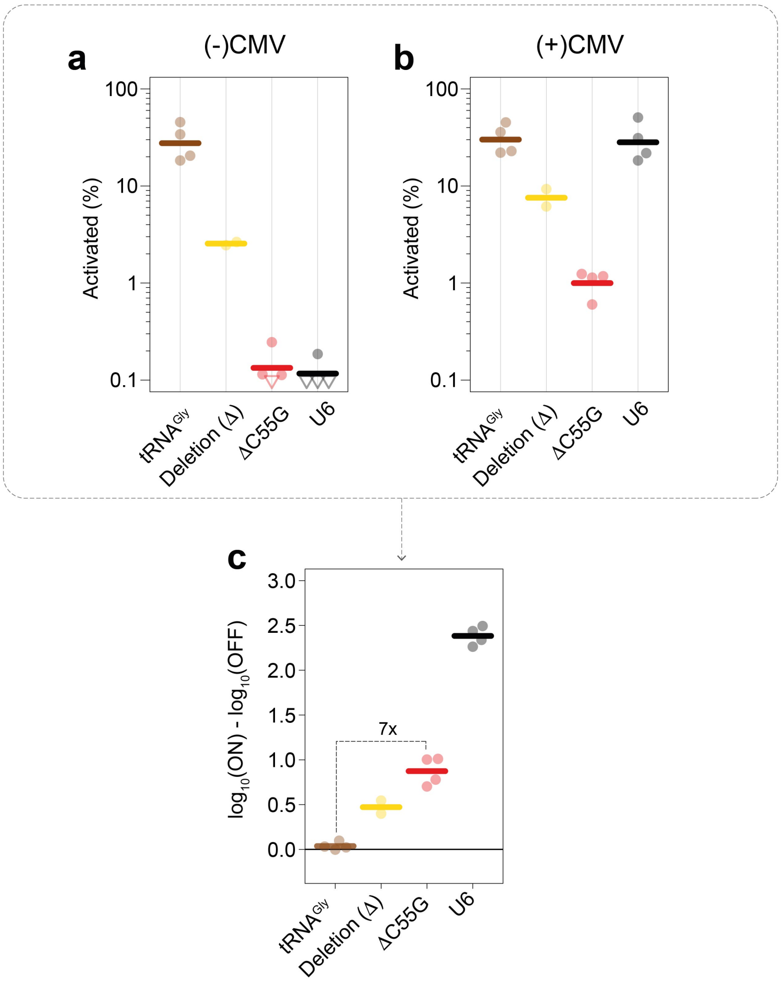

Supplementary Figure 6. The new engineered scaffold is generalizable to other human tRNAs. (a, b) Percentage reporter ECFP^{+} cells within transfected cells for the tRNA^{Gly} constructs with **(a)** and without **(b)** CMV promoters. Points at or below the limit of detection are shown as hollow downward triangles. **(c)** Log$_{10}$(% ECFP^{+} cells) in the ON condition (with CMV promoter) compared to OFF condition (no CMV promoter) for the tRNA^{Gly} constructs (n = 2-4 independent experiments). Thick lines represent mean values. Source data are provided as a Source Data file.

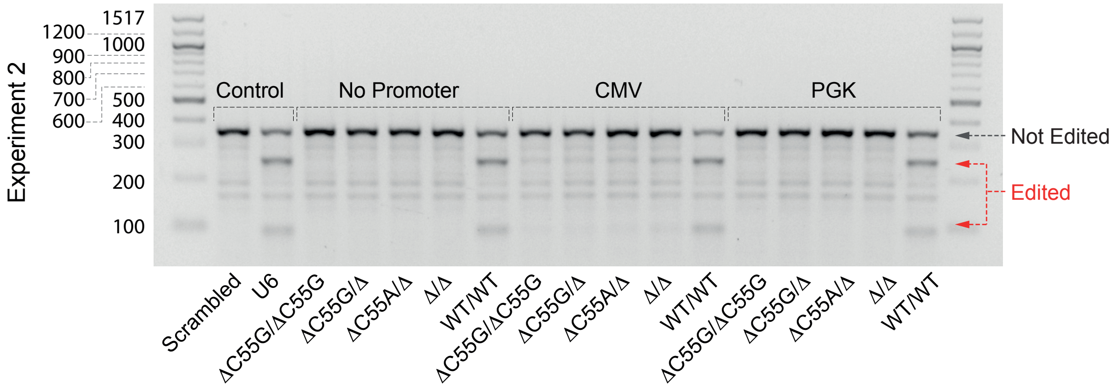

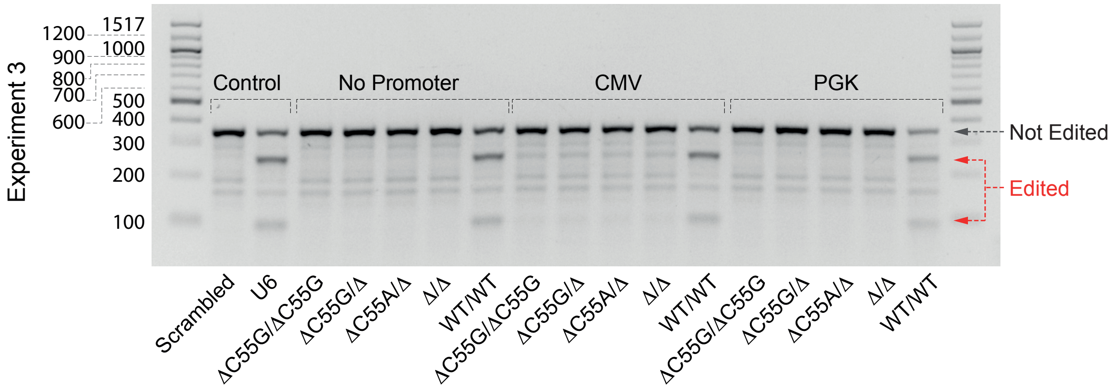

Supplementary Figure 7. T7 endonuclease assay gel images for PDL1 editing. tRNA scaffolds are shown beneath each plot with the 5' variant / 3' variant indicated. A U6 promoter with a scrambled gRNA or with the targetting gRNA are included as controls. Pol-II promoters for each set are noted above their respective lanes. Size in base pairs (bp) are shown next to the left side ladder. Black arrow = the location of uncut (not edited) amplicons; red arrows = digested (edited) amplicons (see Figure 5 for experiment 1 gel).

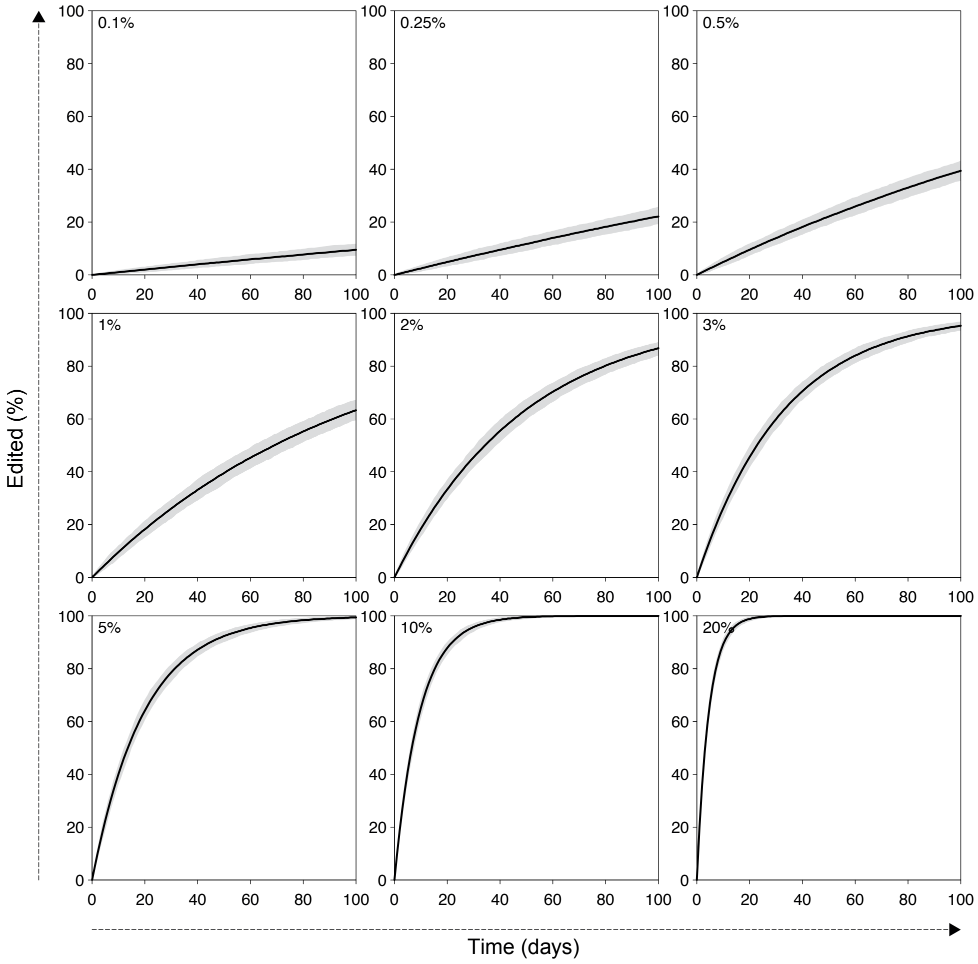

Supplementary Figure 8. Mathematical modeling of indel frequency over time with varying daily editing rates. Results represent 1000 simulated cells each run with a daily binomial probability of editing as indicated in the top left. Time was simulated up to day 100. Lines represent the median value of 1000 replicate simulations, and shaded areas encompass 99% of the total probability distribution.

Supplementary Notes

Modular cloning backbone gBlock sequence

CAGTGCAAGTGCAGGTGCCAGAACATTTCTCTGGGCCCGCCCGGTACCTTCTAGAGGC
TAGGGATGAAGAATAAAAGGGGCGCGCCCCTAGGGAGCGGGGGGCTATAAAAGGGG
GTGGGGGCGTTCGTCCTGCTATCTAGCGTCGCGTTGACCGAGCTCATCGGTGCATGG
GTGGTTCAGTGGTAGAATTCTCGCCTGCCACGCGGGAGGCCCGGGTTCGATTCCCGG
CCCATGCAGGAGACGGACGTCTCCGTTTTAGAGCTAGGCCAACATGAGGATCACCCAT
GTCTGCAGGGCCTAGCAAGTTAAAATAAGGCTAGTCCGTTATCAACTTGGCCAACATGA
GGATCACCCATGTCTGCAGGGCCAAGTGGCACCGAGTCGGTGCAACAAAGCACCAGT
GGTCTAGTGGTGGAATAGTACCCTGCCACGGTACAGACCCGGGTTCGATTCCCGGCTG
GTGCAGGCCGGCCGC

HDV ribozyme sequence

GGCCGGCATGGTCCCAGCCTCCTCGCTGGCGCCGGCTGGGCAACATGCTTCGGCATG
GCGAATGGGAC

mKate intronic sgRNA2.0 gBlock

GCCACCATGGTGTCTAAGGGCGAAGAGCTGATTAAGGAGAACATGCACATGAAGCTGT
ACATGGAGGGCACCGTGAACAACCACCACTTCAAGTGCACATCCGAGGGCGAAGGCA
AGCCCTACGAGGGCACCCAGACCATGAGAATCAAGGTGGTCGAGGGCGGCCCTCTCC
CCTTCGCCTTCGACATCCTGGCTACCAGCTTCATGTACGGCAGCAAAACCTTCATCAAC
CACACCCAGGGCATCCCCGACTTCTTTAAGCAGTCCTTCCCTGAGGTAAGTGGTCCGG
AGACGGACGTCTCCGTTTTAGAGCTAGGCCAACATGAGGATCACCCATGTCTGCAGGG
CCTAGCAAGTTAAAATAAGGCTAGTCCGTTATCAACTTGGCCAACATGAGGATCACCCA
TGTCTGCAGGGCCAAGTGGCACCGAGTCGGTGCTACTAACTCGAGTCTTCTTTTTTTTT
TTCACAGGGCTTCACATGGGAGAGAGTCACCACATACGAAGACGGGGGCGTGCTGAC
CGCTACCCAGGACACCAGCCTCCAGGACGGCTGCCTCATCTACAACGTCAAGATCAGA
GGGGTGAACTTCCCATCCAACGGCCCTGTGATGCAGAAGAAAACACTCGGCTGGGAG
GCCTCCACCGAGATGCTGTACCCCGCTGACGGCGGCCTGGAAGGCAGAAGCGACATG
GCCCTGAAGCTCGTGGGCGGGGGCCACCTGATCTGCAACTTGAAGACCACATACAGAT
CCAAGAAACCCGCTAAGAACCTCAAGATGCCCGGCGTCTACTATGTGGACAGAAGACT
GGAAAGAATCAAGGAGGCCGACAAAGAGACCTACGTCGAGCAGCACGAGGTGGCTGT
GGCCAGATACTGCGACCTCCCTAGCAAACTGGGGCACAAACTTAATTGA

sgRNA2.0, mKate, Splice Donor, Splice Acceptor, Polypyrimidine tract, Branch Point, Guide cloning site

MALAT1 terminator

GATTCGTCAGTAGGGTTGTAAAGGTTTTTCTTTTCCTGAGAAAACAACCTTTTGTTTTCT
CAGGTTTTGCTTTTTGGCCTTTCCCTAGCTTTAAAAAAAAAAAAGCAAAAGACGCTGGTG
GCTGGCACTCCTGGTTTCCAGGACGGGGTTCAAGTCCCTGCGGTGTCTTTGCTT

3' Box terminator

ACTTTCTGGAGTTTCAAAAGTAGACTGTACGCTAAGGGTCATATCTTTTTTTGTTTGGTT
TGTGTCTTGGTTGGCGTCTTAA

Barcode_library

TAACGAGGCGAAGACTAGTGCNNCANNGTNNAGNNNACNNAGCTCACGTCGGAGACG
GACGTCTCCGCCCATGGACNNGANNNTCNNTCNNGANNGTAAATGTCTTCTGGCATTG
C

Variable Bases, Buffer sequence, Dual BsmBI placeholder

*variable bases are 25% of each base at each site

tRNA variant library

CGTCTCtcgtcGGCTCGTNNGTCTNNNNNNNTGATTCTCGCTTAGGGTGCGAGAGGTCCC GGGNNNNNNNCCCGGACGAGCCCtGAGACG

$tRNA^{Pro}$, Variable Bases

*variable bases are 82% of the wild-type base and 6% each of the three other possible bases at that location

Supplementary Tables

Oligo Pair	Forward	Reverse
1	GCTAGCGGTACCGGTACTTGGAGCGGCCGC	GGCGCCGGTACCCGATAGAGAAATGTTCTGGCACCTG
2	TTTTGCTCACATGTGCATGCGGAGCGGCCGCAATAAAATATCT	CTAGGGCGCTGGCAAGTGTA
3	CAGTGCAAGTGCAGGTGCCA	GCGGCCGGCCTGCAC
4	CCCGGCTGGTGCAGGCCGGCCGCTTC	GCCACCTGACGTCCCTGCAGGCTCGAGGGATCCTTATCGATTTTACC
5	ACGGAGACGTCGAATGTGTGTCAGTTAGGGTG	TACGTCTCGAGTTAGCTCACTCATTAGGCAC
6	TGCTTACATGTGGAATGTGTGTCAGTTAG	CTAACCTCGAGCAAGCTCTAGCTAGAGGTCG
7	CGAGAAGACCTGTTTTAGAGCTAG	GAAGCGGCCGGCCAAAAAAGCACCGACTCG
8	ACGTTGGCGCGCCCGAGCATTAGTTCATAGCCCATATATGG	GCAACGAGCTCGACCGGTGGATCTG
9	AAGTAGAGCTCGCCACCATGGTGTCTAAGG	AATGAGGCCGGCCTCAATTAAGTTTGTGCCCCA
10	TGCCAGAAGACATGGAGACGGACGTCTCCGTTTTAG	GAGGCGAAGACTAGCACCGACTCGGTGCCA
11	GAGGCGAAGACTAGTGCGGCCGGCATGGTCCC	AAGTAGGCCGGCCGTCCCATTCGCCATGCC
12	ATTTAGAAGACAACGCGCCGTAAGTCGGAGTACTGTCCTGTTTTAGAGC	GAGGCGAAGACTAGCACCGACTCGGTGCCA
13	ATTTAGAAGACAACCGGTGTAAGTCGGAGTACTGTCCTGTTTTAGAGC	"
14	TGCCAGAAGACATGTGCGATTCGTCAGTAGGGTTGTAAAGG	TGCCAGAAGACATTCGAGAAGCAAAGACACCGCAGG
15	ATTTAGAAGACAAGTGCGGATCCACTTTCTGGAGTTTCAAAAG	ATTTAGAAGACAATCGAGTTAAGACGCCAACCAAG
16	TGGATGGCGCGCCTAGTGAACCGTCAGATCCAC	ACAGAGAGCTCAGTTATTAGGTCCCTCGACG
17	ACGTTGGCGCGCCCGAGCATTAGTTCATAGCCCATATATGG	ACAGAGAGCTCAGTTATTAGGTCCCTCGACG
18	AGCACGAGCTCGTAAGTCGGAGTACTGTCCTGTTTTAGAG	TCGAAGACCCGCACCGACTCGGTGCC
19	TAAGAAGACTAGTAAGTCGGAGTACTGTCCTGTTTTAGAG	GAAGCGGCCGGCCAAAAAAGC
20	TAACGAGGCGAAGACTAGTGC	GCAATGCCAGAAGACATTTAC
21	TAACGTCTCTCGTCGGCTCG	TAACGTCTCAGGGCTCGTCC
22	ATGATGCAACTCGTAGGACAGGTG	CATGCACCTGTCCTACGAGTTGCA
23	CATGCCAACATGTCTTCGAATTCGAAGACTAGGACGGCCGG	CCGTCCTAGTCTTCGAATTCGAAGACATGTTGG
24	GCCCGGAGACGGGAATTCACGTCTCCGTTTTAGAGCTAGAAATAGCAAGTTAAAATA	GCCTTATTTTAACTTGCTATTTCTAGCTCTAAAACGGAGACGTGAATTCCCGTCTCC
25	AGGCTAGTCCGTTATCAACTTGAAAAAGTGGCACCGAGTCGGTGC	GGTTGCACCGACTCGGTGCCACTTTTTCAAGTTGATAACGGACTA
26	AAGGAGAAGACTAGCCCGGAGACGGACGTCTCCGTTTTAGAG	AAGGAGAAGACAAGGTTGCACCGACTCGGTGCCA
27	GCCCTGGGAAGCAGAGGCAAAGGG	AAACCCCTTTGCCTCTGCTTCCCA
28	GCCCTCTTCTTGGTATGGTCCTAA	AAACTTAGGACCATACCAAGAAGA
29	AAGGAGAAGACAACTAGCGTTACATAACTTACGGTAAATG	AAGGAGAAGACAAGTTGGACCGGTGGATCTGACG
30	AAGGAGCTAGCCCGGGTAGGGGAGGC	AAGGAGAAGACAACCGGCACGCGTAAGCTTGG
31	TTAACGCTAGCGGCAGTGGAGAGGGCAGAG	TAGTTAGAATTCTCAGGCACCGGGC
32	TTAAAGAAGACTTCCCAATGGCCAAGTTGACCAGTGCC	AGTTAGAATTCTCAGTCCTGCTCCTCGGCC
33	CTAGCGGCAGTGGAGAGGGCAGAGGAAGTCTGCTAACATGCGGTGACGTCGAGGAGAATCCTGG	TGGGCCAGGATTCTCCTCGACGTCACCGCATGTTAGCAGACTTCCTCTGCCCTCTCCACTGCCG
34	CTAGCTAAGTGTACAAGTAAG	AATTCTTACTTGTACACTTAG
35	CATGTGATATCACAAATGGCT	CTAGAGCCATTTGTGATATCA

Supplementary Table 1. Miscellaneous oligos used in cloning.

Oligo Name	Forward	Reverse
Hist1h3h term	GTGCGTCTCAAGGACTCACTGATTACATACCCAAAGGCTCTTTTCAGAGCCACCCACATGCGCGCTGAAAAGATCTGTTTCTCTC	TCGAGAGAGAAACAGATCTTTTCAGCGCGCATGTGGGTGGCTCTGAAAAGAGCCTTTGGGTATGTAATCAGTGAGTCCTTGAGAC
min-pA	GTGCTCCTTTATCTTCATTGGATCCGTGTGTTGGTTTTTTGTGTGCGGCCCGTCTACC	TCGAGGTAGACGGGCCGCACACAAAAAACCAACACACGGATCCAATGAAGATAAAGGA
HH_CTS2	CACTTACCTGATGAGTCCGTGAGGACGAAACGAGTAAGCTCGTCGTAAGTCGGAGTACTGTCCT	AAACAGGACAGTACTCCGACTTACGACGAGCTTACTCGTTTCGTCCTCACGGACTCATCAGGTAAGTGAGCT
CTS2-tRNAGly	TGCAGTAAGTCGGAGTACTGTCCT	AAACAGGACAGTACTCCGACTTAC
CTS2-tRNAPro	GCCCGTAAGTCGGAGTACTGTCCT	"
CTS2-tRNAGln	ACCTGTAAGTCGGAGTACTGTCCT	"
CTS2-intron	GTCCGTAAGTCGGAGTACTGTCCT	"
CTS2-Csy4	CAGCGTAAGTCGGAGTACTGTCCT	"
iBlueRemover	TCGAAATCGCCTGCAGGGATACACGT	GTATCCCTGCAGGCGATT
BpiI_placeholder	GTGCGGGTCTTCGAGAAGACCT	TTACAGGTCTTCTCGAAGACCC
hu-tRNAGly	CATCGGTGCATGGGTGGTTCAGTGGTAGAATTCTCGCCTGCCACGCGGGAGGCCCGGGTTCGATTCCCGGCCCA	TGCATGGGCCGGGAATCGAACCCGGGCCTCCCGCGTGGCAGGCGAGAATTCTACCACTGAACCACCCATGCaccgatGAGCT
rice-tRNAGly	CAACAAAGCACCAGTGGTCTAGTGGTGGAATAGTACCCTGCCACGGTACAGACCCGGGTTCGATTCCCGGCTGG	TGCACCAGCCGGGAATCGAACCCGGGTCTGTACCGTGGCAGGGTACTATTCCACCACTAGACCACTGGTGCTTTGTTGAGCT
fly-tRNAGly Part 1	CGGGCTTTGAGTGTGTGTAGACATCAAGCATCGGTGGTTCAGTGGTAGAATG	CGAGCATTCTACCACTGAACCACCGATGCTTGATGTCTACACACACTCAAAGCCCGAGCT
fly-tRNAGly Part 2	CTCGCCTGCCACGCGGGCGGCCCGGGTTCGATTCCCGGCCGA	TGCATCGGCCGGGAATCGAACCCGGGCCGCCCGCGTGGCAGG
hu-tRNAGln	CGGTTCCATGGTGTAATGGTTAGCACTCTGGACTCTGAATCCAGCGATCCGAGTTCAAATCTCGGTGGA	AGGTTCCACCGAGATTTGAACTCGGATCGCTGGATTCAGAGTCCAGAGTGCTAACCATTACACCATGGAACCGAGCT
hu-tRNAPro Part 1	CGGCTCGTTGGTCTAGGGGTATGATTCTCGCTTAGGGTGC	CCTAAGCGAGAATCATACCCCTAGACCAACGAGCCGAGCT
hu-tRNAPro Part 2	GAGAGGTCCCGGGTTCAAATCCCGGACGA	GGGCTCGTCCGGGATTTGAACCCGGGACCTCTCGCAC
G9C hu-tRNAPro Part 1	CGGCTCGTTCGTCTAGGGGTATGATTCTCGCTTAGGGTGC	CCTAAGCGAGAATCATACCCCTAGACGAACGAGCCGAGCT
G16C hu-tRNAPro Part 1	CGGCTCGTTGGTCTAGCGGTATGATTCTCGCTTAGGGTGC	CCTAAGCGAGAATCATACCGCTAGACCAACGAGCCGAGCT
G17T hu-tRNAPro Part 1	CGGCTCGTTGGTCTAGGTGTATGATTCTCGCTTAGGGTGC	CCTAAGCGAGAATCATACACCTAGACCAACGAGCCGAGCT
G17A/A20G hu-tRNAPro Part 1	CGGCTCGTTGGTCTAGGAGTGTGATTCTCGCTTAGGGTGC	CCTAAGCGAGAATCACACTCCTAGACCAACGAGCCGAGCT
G18T hu-tRNAPro Part 1	CGGCTCGTTGGTCTAGGGTTATGATTCTCGCTTAGGGTGC	CCTAAGCGAGAATCATAACCCTAGACCAACGAGCCGAGCT
T54C|A58C hu-tRNAPro Part 2	GAGAGGTCCCGGGTCCAACTCCCGGACGA	GGGCTCGTCCGGGAGTTGGACCCGGGACCTCTCGCAC
C55A hu-tRNAPro Part 2	GAGAGGTCCCGGGTTAAAATCCCGGACGA	GGGCTCGTCCGGGATTTTAACCCGGGACCTCTCGCAC
C55G hu-tRNAPro Part 2	GAGAGGTCCCGGGTTGAAATCCCGGACGA	GGGCTCGTCCGGGATTTCAACCCGGGACCTCTCGCAC
A56T hu-tRNAPro Part 2	GAGAGGTCCCGGGTTCTAATCCCGGACGA	GGGCTCGTCCGGGATTAGAACCCGGGACCTCTCGCAC
A58T|T59A hu-tRNAPro Part 2	GAGAGGTCCCGGGTTCAATACCCGGACGA	GGGCTCGTCCGGGTATTGAACCCGGGACCTCTCGCAC
A56T|T59C hu-tRNAPro Part 2	GAGAGGTCCCGGGTTCTAACCCCGGACGA	GGGCTCGTCCGGGGTTAGAACCCGGGACCTCTCGCAC
del/C55A hu-tRNAPro	CGGCTCGTTGGGAGGTCCCGGGTTAAAATCCCGGACGA	GGGCTCGTCCGGGATTTTAACCCGGGACCTCCCAACGAGCCGAGCT
del/T54C/A58C hu-tRNAPro	CGGCTCGTTGGGAGGTCCCGGGTCCAACTCCCGGACGA	GGGCTCGTCCGGGAGTTGGACCCGGGACCTCCCAACGAGCCGAGCT
del/G9C/A56T hu-tRNAPro	CGGCTCGTTCGGAGGTCCCGGGTTCTAATCCCGGACGA	GGGCTCGTCCGGGATTAGAACCCGGGACCTCCGAACGAGCCGAGCT
del/C55G hu-tRNApro	CGGCTCGTTGGGAGGTCCCGGGTTGAAATCCCGGACGA	GGGCTCGTCCGGGATTTCAACCCGGGACCTCCCAACGAGCCGAGCT
del/C55G hu-tRNAGly	CATCGGTGCATGGGTGGGAGGCCCGGGTTGGATTCCCGGCCCA	TGCATGGGCCGGGAATCCAACCCGGGCCTCCCACCCATGCACCGATGAGCT
del hu-tRNAGly	CATCGGTGCATGGGTGGGAGGCCCGGGTTCGATTCCCGGCCCA	TGCATGGGCCGGGAATCGAACCCGGGCCTCCCACCCATGCACCGATGAGCT

Supplementary Table 2. Annealed oligonucleotides used in cloning experiments.

Forward Primer Name	Forward Primer Sequence	Reverse Primer Name	Reverse Primer Sequence
Amp_Pro_F	ATCCAGGCCGGCCGGCTCGTTGGTCTAGGGG	Amp_Pro_R	AACGTGGCCGGCCGGGCTCGTCCGGG
Amp_Pro_G9C_F	ATCCAGGCCGGCCGGCTCGTTCGTCTAGGGG	Amp_Pro_R	AACGTGGCCGGCCGGGCTCGTCCGGG
Amp_Pro_del_F	ATCCAGGCCGGCCGGCTCGTTGGGAGGTC	Amp_Pro_R	AACGTGGCCGGCCGGGCTCGTCCGGG
Amp_Pro_delG9C_F	ATCCAGGCCGGCCGGCTCGTTCGGAGGTC	Amp_Pro_R	AACGTGGCCGGCCGGGCTCGTCCGGG
Amp_ProG17A_F	ATCCAGGCCGGCCGGCTCGTTGGTCTAGGAG	Amp_Pro_R	AACGTGGCCGGCCGGGCTCGTCCGGG
Amp_Pro_del_F	ATCCAGGCCGGCCGGCTCGTTGGGAGGTC	Amp_Pro_R	AACGTGGCCGGCCGGGCTCGTCCGGG
Amp_ProG17T_F	ATCCAGGCCGGCCGGCTCGTTGGTCTAGGTG	Amp_Pro_R	AACGTGGCCGGCCGGGCTCGTCCGGG
Amp_ProG18T_F	ATCCAGGCCGGCCGGCTCGTTGGTCTAGGGT	Amp_Pro_R	AACGTGGCCGGCCGGGCTCGTCCGGG
Amp_Gly_del-F	ATCCAGGCCGGCCATCGGTGCATGGGTGG	Amp_Gly_del-R	AACGTGGCCGGCCTGCATGGGCCGGGA
minCsy4-CTS2-F	TGCCAGAGCTCCTGCCGTATAGGCAGCGTAAGTCGGAGTACTGTCCTGTTTTAGAGC	minCys4-sgRNA-R	TGCCAGGCCGGCCGCTGCCTATACGGCAGGCACCGACTCGGTGCCA
FullCsy4-CTS2-F	AAAAAGAGCTCGTTCACTGCCGTATAGGCAGCTAAGAAAGTAAGTCGGAGTACTGTCCTGTTTTAGAGC	FullCsy4-sgRNA-R	AAAAAGGCCGGCCTTTCTTAGCTGCCTATACGGCAGTGAACGCACCGACTCGGTGCCA
SacI-Csy4nano-plch-F	TGCCAGAGCTCACTGCCGTATAGGCAGCGGAGACGGACGTCTCCGTTTTAG	Csy4nano-sgRNA-R	TTAAAGGCCGGCCGCTGCCTATACGGCAGTGCACCGACTCGGTGCCA
BpiI-CAAC-5p-tRNApro-F	AAGGAGAAGACTACAACGGCTCGTTGGGAGGTC	BpiI-GCCC-5p-tRNApro-R	AAGGAGAAGACAAGGGCTCGTCCGGGATTT
BpiI-14bpBuf-3p-deltRNApro-F	AAGGAGAAGACAAAACCAGTTTGTGTCGGCTCGTTGGGAGGT	3p-del-tRNApro-R	AAGGAGAAGACTAGTCCCGGGCTCGTCCGGGA
BpiI-CAAC-5p-WTtRNApro-F	AAGGAGAAGACTACAACGGCTCGTTGGTCTAGGGG	BpiI-GCCC-5p-tRNApro-R	AAGGAGAAGACAAGGGCTCGTCCGGGATTT
BpiI-14bpBuf-3p-WTtRNApro-F	AAGGAGAAGACAAAACCAGTTTGTGTCGGCTCGTTGGTCTAGGGG	3p-del-tRNApro-R	AAGGAGAAGACTAGTCCCGGGCTCGTCCGGGA

Supplementary Table 3. Primers used for tRNA and Csy4 amplification.

Forward Primer Name	Forward Primer Sequence	Reverse Primer Name	Reverse Primer Sequence
cRT-sgRNA1_LIB_F	ACACTCTTTCCCTACACGACGCTCTTCCGATCTGGCTAGTCCGTTATCAACT	cRT-CTS2_LIB_R	GTGACTGGAGTTCAGACGTGTGCTCTTCCGATCTAGGACAGTACTCCGACTTAC
cRT-sgRNA_nest_F	TAGCAAGTTAAAATAAGGCTAGT	cRT-CTS2_nest_R	CTAGCTCTAAAACAGGACAGT
CTS2_qPCR-F	AGTCGGAGTACTGTCCTGTT	sgRNA_qPCR_common-R	GGACTAGCCTTATTTTAACTTGCT
dCas9_qPCR-F	CCCAAGAGGAACAGCGATAAG	dCas9_qPCR-R	CCACCACCAGCACAGAATAG
cRT-sgRNA1_v2_F	GGCTAGTCCGTTATCAACT	cRT-CTS2_R	AGGACAGTACTCCGACTTAC

Supplementary Table 4. Primers used for RNA circularization assays and qPCR.

Sample Type (cRNA/pDNA)	Replicate	CMV (+/-)	Forward Index Name	Forward Index Sequence	Reverse Index Name	Reverse Index Sequence
circRNA	1	-	D508	AATGATACGGCGACCACCGAGATCTACACGTACTGACACACTCTTTCCCTACACGAC	D701	CAAGCAGAAGACGGCATACGAGATCGAGTAATGTGACTGGAGTTCAGACGTGT
circRNA	2	-	D508	AATGATACGGCGACCACCGAGATCTACACGTACTGACACACTCTTTCCCTACACGAC	D702	CAAGCAGAAGACGGCATACGAGATTCTCCGGAGTGACTGGAGTTCAGACGTGT
circRNA	3	-	D508	AATGATACGGCGACCACCGAGATCTACACGTACTGACACACTCTTTCCCTACACGAC	D703	CAAGCAGAAGACGGCATACGAGATAATGAGCGGTGACTGGAGTTCAGACGTGT
circRNA	1	+	D508	AATGATACGGCGACCACCGAGATCTACACGTACTGACACACTCTTTCCCTACACGAC	D704	CAAGCAGAAGACGGCATACGAGATGGAATCTCGTGACTGGAGTTCAGACGTGT
circRNA	2	+	D508	AATGATACGGCGACCACCGAGATCTACACGTACTGACACACTCTTTCCCTACACGAC	D705	CAAGCAGAAGACGGCATACGAGATTTCTGAATGTGACTGGAGTTCAGACGTGT
circRNA	3	+	D508	AATGATACGGCGACCACCGAGATCTACACGTACTGACACACTCTTTCCCTACACGAC	D706	CAAGCAGAAGACGGCATACGAGATACGAATTCGTGACTGGAGTTCAGACGTGT
pDNA	1	-	D508	AATGATACGGCGACCACCGAGATCTACACGTACTGACACACTCTTTCCCTACACGAC	D707	CAAGCAGAAGACGGCATACGAGATAGCTTCAGGTGACTGGAGTTCAGACGTGT
pDNA	2	-	D508	AATGATACGGCGACCACCGAGATCTACACGTACTGACACACTCTTTCCCTACACGAC	D708	CAAGCAGAAGACGGCATACGAGATGCGCATTAGTGACTGGAGTTCAGACGTGT
pDNA	3	-	D508	AATGATACGGCGACCACCGAGATCTACACGTACTGACACACTCTTTCCCTACACGAC	D709	CAAGCAGAAGACGGCATACGAGATCATAGCCGGTGACTGGAGTTCAGACGTGT
pDNA	1	+	D508	AATGATACGGCGACCACCGAGATCTACACGTACTGACACACTCTTTCCCTACACGAC	D710	CAAGCAGAAGACGGCATACGAGATTTCGCGGAGTGACTGGAGTTCAGACGTGT
pDNA	2	+	D508	AATGATACGGCGACCACCGAGATCTACACGTACTGACACACTCTTTCCCTACACGAC	D711	CAAGCAGAAGACGGCATACGAGATGCGCGAGAGTGACTGGAGTTCAGACGTGT
pDNA	3	+	D508	AATGATACGGCGACCACCGAGATCTACACGTACTGACACACTCTTTCCCTACACGAC	D712	CAAGCAGAAGACGGCATACGAGATCTATCGCTGTGACTGGAGTTCAGACGTGT

Supplementary Table 5. Deep sequencing indexes.
