# Supplementary material for: Decoupling tRNA promoter and processing activities enables specific Pol-II Cas9 guide RNA expression
Source: Nat Commun. 2019 Apr 2;10:1490. doi: 10.1038/s41467-019-09148-3 (PMC6445147; doi:10.1038/s41467-019-09148-3)
Supplement: Supplementary file 3 — Reporting Summary [file 41467_2019_9148_MOESM3_ESM.pdf]

nature research

Corresponding author(s): Tudor A Fulga, David JHF Knapp

Last updated by author(s): Feb 15, 2019

Reporting Summary

Nature Research wishes to improve the reproducibility of the work that we publish. This form provides structure for consistency and transparency in reporting. For further information on Nature Research policies, see Authors & Referees and the Editorial Policy Checklist.

Statistics

For all statistical analyses, confirm that the following items are present in the figure legend, table legend, main text, or Methods section.

n/a	Confirmed	
☐	☒	The exact sample size (n) for each experimental group/condition, given as a discrete number and unit of measurement
☐	☒	A statement on whether measurements were taken from distinct samples or whether the same sample was measured repeatedly
☐	☒	The statistical test(s) used AND whether they are one- or two-sided<br>*Only common tests should be described solely by name; describe more complex techniques in the Methods section.*
☒	☐	A description of all covariates tested
☐	☒	A description of any assumptions or corrections, such as tests of normality and adjustment for multiple comparisons
☐	☒	A full description of the statistical parameters including central tendency (e.g. means) or other basic estimates (e.g. regression coefficient) AND variation (e.g. standard deviation) or associated estimates of uncertainty (e.g. confidence intervals)
☒	☐	For null hypothesis testing, the test statistic (e.g. F, t, r) with confidence intervals, effect sizes, degrees of freedom and P value noted<br>*Give P values as exact values whenever suitable.*
☐	☒	For Bayesian analysis, information on the choice of priors and Markov chain Monte Carlo settings
☒	☐	For hierarchical and complex designs, identification of the appropriate level for tests and full reporting of outcomes
☒	☐	Estimates of effect sizes (e.g. Cohen's d, Pearson's r), indicating how they were calculated

Our web collection on statistics for biologists contains articles on many of the points above.

Software and code

Policy information about availability of computer code

Data collection	FACSDiva (version 8.0.1) was used for the collection of flow cytometry data. ImageLab (version 5.2.1) was used for gel imaging. Bio-Rad CFX Manager (version 3.1) was used for qPCR data acquisition.
Data analysis	R (version 3.2.1 and 3.4.1) was used for all data analysis. The R package 'BayesianFirstAid' (version 0.1) was used for significance testing, 'flowCore' (version 1.44.2) together with custom scripts for data plotting used for flow cytometric analysis, and 'ShortRead' (version 1.26.0), 'Biostrings' (version 2.36.4), and 'plyr' (version 1.8.4) were used together with custom scripts for the analysis of the tRNA library sequencing data. GelAnalyzer2010a was used for gel analysis. Snapgene (version 4.2.11) was used to plan molecular cloning strategies.

For manuscripts utilizing custom algorithms or software that are central to the research but not yet described in published literature, software must be made available to editors/reviewers. We strongly encourage code deposition in a community repository (e.g. GitHub). See the Nature Research guidelines for submitting code & software for further information.

Data

Policy information about availability of data

All manuscripts must include a data availability statement. This statement should provide the following information, where applicable:

- Accession codes, unique identifiers, or web links for publicly available datasets
- A list of figures that have associated raw data
- A description of any restrictions on data availability

Raw HTS data (Fastq files) have been deposited into the Sequence Read Archive (SRA). SRA accession: PRJNA521493. All other raw data that is not found in the supplementary information is available from the corresponding author upon reasonable request. Relevant plasmids described in this study are available from Addgene (http://www.addgene.org/Tudor_Fulga/).

Field-specific reporting

Please select the one below that is the best fit for your research. If you are not sure, read the appropriate sections before making your selection.

☒ Life sciences ☐ Behavioural & social sciences ☐ Ecological, evolutionary & environmental sciences

For a reference copy of the document with all sections, see nature.com/documents/nr-reporting-summary-flat.pdf

Life sciences study design

All studies must disclose on these points even when the disclosure is negative.

Sample size	As many experiments as logistically possible were performed to ensure that functional differences between tRNA variants could be assessed.
Data exclusions	For sequencing data, only reads which could be unambiguously assigned to a barcode-tRNA variant pair were included as this was required for accurate measurement. No other data was excluded.
Replication	Data from all replicate experiments is included in the present manuscript and was successful in all cases.
Randomization	Randomization is not relevant as all tests were either unbiased screens, or functional tests to determine the effects of specific variants on tRNA function/gRNA release.
Blinding	Blinding was not relevant as as all tests were either unbiased screens, or functional tests to determine the effects of specific variants on tRNA function/gRNA release.

Reporting for specific materials, systems and methods

We require information from authors about some types of materials, experimental systems and methods used in many studies. Here, indicate whether each material, system or method listed is relevant to your study. If you are not sure if a list item applies to your research, read the appropriate section before selecting a response.

Materials & experimental systems

n/a	Involved in the study	
☐	☒	Antibodies
☐	☒	Eukaryotic cell lines
☒	☐	Palaeontology
☒	☐	Animals and other organisms
☒	☐	Human research participants
☒	☐	Clinical data

Methods

n/a	Involved in the study	
☒	☐	ChIP-seq
☐	☒	Flow cytometry
☒	☐	MRI-based neuroimaging

Antibodies

Antibodies used	CD271 (clone C40-1457) V450 (BD Biosciences, cat #: 562123)
Validation	Antibody was used to stain HEK239T with and without a NGFR specific gRNA and Cas9 transcriptional activator to verify specific staining only in the case of gRNA presence.

Eukaryotic cell lines

Policy information about cell lines

Cell line source(s)	ATCC
Authentication	No authentication was performed as cell identity is immaterial to the results of the study.
Mycoplasma contamination	Mycoplasma testing was performed at least every 6 months using either a VenorGeM® Mycoplasma Kit (Minerva Biolabs) according to manufacturer protocols, or using a set of primers from REF29,30.
Commonly misidentified lines (See ICLAC register)	HEK239T were used due to simplicity of transfection.

Flow Cytometry

Plots

Confirm that:

☒ The axis labels state the marker and fluorochrome used (e.g. CD4-FITC).

☒ The axis scales are clearly visible. Include numbers along axes only for bottom left plot of group (a 'group' is an analysis of identical markers).

☒ All plots are contour plots with outliers or pseudocolor plots.

☒ A numerical value for number of cells or percentage (with statistics) is provided.

Methodology

Sample preparation	Cells were harvested using 0.05% Trypsin-EDTA (Thermo Fisher Scientific), washed once in PBS + 2% FBS.<br>For antibody staining: Where drug selection was used, cells were then resuspended in 50 µl of PBS + 2% FBS + 0. 25 µl Zombie RedTM Fixable Viability Dye (Biolegend) and incubated on ice for 15 minutes in the dark. These were then washed once in 1 ml PBS + 2% FBS. Samples were then stained in 50 µl PBS + 2% FBS + 0.5 µl mouse anti-human NGFR aka CD271 (clone C40-1457) V450 (BD Biosciences) for 30 minutes on ice in the dark. Cells were washed once with 1 ml PBS + 2% FBS, filtered then analyzed by flow cytometry.<br>For fluorophore analysis only: Cells were filtered then analyzed by flow cytometry.
Instrument	Flow cytometry was performed using either a BD LSRFortessa cell analyzer or BD LSRII flow cytometer. ECFP was measured following 405 nm excitation with a 450/50 bandpass filter. EGFP was measured using a 488 nm excitation with a 530/30 (Fortessa) or 525/50 (LSRII) bandpass filter. iBlue was measured using a 640 nm excitation with a 670/14 bandpass filter. mCherry and mKate (for the intron release system) were measured with a 561 (Fortessa) of 532 nm (LSRII) excitation with a 610/20 bandpass filter.
Software	BD FACSDiva version 8.0.1
Cell population abundance	Sorting was not performed.
Gating strategy	Cells were manually gated for positivity of transfection controls. Positive gates for final reporters (NGFR staining or ECFP reporter expression) were set using scrambled gRNA or no promoter controls.

☒ Tick this box to confirm that a figure exemplifying the gating strategy is provided in the Supplementary Information.
